# Inhibition of BET Proteins Regulates Fcγ Receptor Function and Reduces Inflammation in Rheumatoid Arthritis

**DOI:** 10.3390/ijms24087623

**Published:** 2023-04-21

**Authors:** Divya Shankar, Giovanna Merchand-Reyes, Nathaniel J. Buteyn, Ramasamy Santhanam, Huiqing Fang, Krishan Kumar, Xiaokui Mo, Latha P. Ganesan, Wael Jarjour, Jonathan P. Butchar, Susheela Tridandapani

**Affiliations:** 1 College of Medicine, The Ohio State University, Columbus, OH 43210, USA; 2 Department of Internal Medicine, The Ohio State University, Columbus, OH 43210, USA; 3 Van Andel Institute, Grand Rapids, MI 49503, USA; 4 Department of Biomedical Informatics, The Ohio State University, Columbus, OH 43210, USA

**Keywords:** FcγR, rheumatoid arthritis, monocytes, BET inhibition

## Abstract

Overactivation of immune responses is a hallmark of autoimmune disease pathogenesis. This includes the heightened production of inflammatory cytokines such as Tumor Necrosis Factor α (TNFα), and the secretion of autoantibodies such as isotypes of rheumatoid factor (RF) and anticitrullinated protein antibody (ACPA). Fcγ receptors (FcγR) expressed on the surface of myeloid cells bind Immunoglobulin G (IgG) immune complexes. Recognition of autoantigen-antibody complexes by FcγR induces an inflammatory phenotype that results in tissue damage and further escalation of the inflammatory response. Bromodomain and extra-terminal protein (BET) inhibition is associated with reduced immune responses, making the BET family a potential therapeutic target for autoimmune diseases such as rheumatoid arthritis (RA). In this paper, we examined the BET inhibitor PLX51107 and its effect on regulating FcγR expression and function in RA. PLX51107 significantly downregulated expression of FcγRIIa, FcγRIIb, FcγRIIIa, and the common γ-chain, FcϵR1-γ, in both healthy donor and RA patient monocytes. Consistent with this, PLX51107 treatment attenuated signaling events downstream of FcγR activation. This was accompanied by a significant decrease in phagocytosis and TNFα production. Finally, in a collagen-induced arthritis model, PLX51107-treatment reduced FcγR expression in vivo accompanied by a significant reduction in footpad swelling. These results suggest that BET inhibition is a novel therapeutic approach that requires further exploration as a treatment for patients with RA.

## 1. Introduction

Rheumatoid arthritis (RA) is a disease characterized by systemic inflammation, mainly of the joints but occasionally in multiple other organs, that can lead, if untreated, to significant morbidity as well as mortality [1,2,3]. Approximately 1% of people worldwide develop RA, and current treatments involve the use of immunosuppressants that specifically target proinflammatory cell-mediated inflammation [1].

The presence of autoantibodies has been used to identify the development of the disease [4]. In RA, rheumatoid factor (RF) as well as the anti-citrullinated peptide antibody (ACPA) are often of the IgG subtype, and, as such, can be recognized by Fcγ receptors (FcγR) on immune cells such as monocytes and macrophages [1,4,5]. Notably, it has been demonstrated that monocytes from individuals with active RA display higher surface expression of FcγRIa and FcγRIIa than healthy controls [5]. Further, the blocking of FcγRIIa by specific F(ab)’_2_ fragments was able to mitigate RA-mediated inflammation and significantly reduced FcγR-mediated production of reactive oxygen species (ROS) [6]. In addition, macrophages and monocytes have been identified as two of the primary immune cell infiltrates in synovium [7]. Engagement with autoantibody immune complexes in conjunction with other FcγR-mediated inflammatory responses contributes to the autoimmune inflammation that results in damage to tissue and joints [7].

Inhibitors of tumor necrosis factor alpha (TNFα) and methotrexate are major components of current RA treatment [8]. For patients unresponsive to TNFα inhibitors, the anti-CD20, rituximab, is utilized to deplete B cells in the peripheral blood and synovial regions [9]. However, there is still a proportion of RA patients resistant to different treatments known as difficult-to-treat RA [10]. Thus, it is imperative to find novel therapies that effectively block inflammation in these patients.

Bromodomain and extra-terminal (BET) proteins are key regulators of transcription that are characterized by two bromodomains and an extra-terminal domain [11]. BET proteins interact with acetylated lysine residues on histones and regulate transcription. Inhibition of this regulatory function has shown significant potential in reducing autoimmune inflammation by downregulating expression of key proinflammatory cytokines, including interleukin 6 (IL-6) and interferon-γ (IFNγ), making BET inhibition potentially therapeutic for RA [12].

Although the role of BET inhibition in dampening inflammatory responses is well established, little is known about their potential effects on antibody-mediated responses. Here, we showed that BET inhibition downregulates expression of FcγRIIa, FcγRIIb, FcγRIIIa, and the common gamma chain FcεR1γ, while upregulating FcγRIa. This corresponded to reduced antibody-mediated responses, including FcγR-mediated signaling, phagocytosis, and TNFα production. Consistent with this, a mouse model of collagen-induced arthritis showed that BET inhibition significantly reduced FcγR expression in the spleen and, most importantly, a clinical response in this murine model. Taken together, these results suggest that BET inhibition affects not only general inflammatory responses but also FcγR expression and function.

## 2. Results and Discussionγ

### 2.1. BET Inhibition Reduces Fcγ Receptor Expression in Peripheral Blood Monocytes

**Figure 1:**
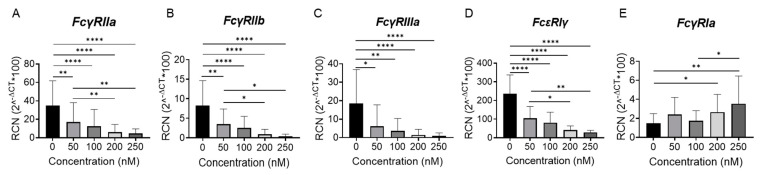
BET inhibition reduces FcγR transcript in human monocytes. Healthy donor CD14+ monocytes were cultured for 24 h with PLX51107 (50, 100, 200, 250 nM) or with vehicle control (DMSO). Transcript expression was analyzed by real-time PCR for (**A**) FcγRIIa, (**B**) FcγRIIb, (**C**) FcγRIIIa, (**D**) FcεRIγ, and (**E**) FcγRIa. (n = 8). * *p* ≤ 0.05, ** *p* ≤ 0.01, **** *p* ≤ 0.0001 respectively.

Inhibition of BET proteins has been well documented to suppress inflammation through interference with both the inflammatory gene and TNFα-induced gene transcription [12]. However, the impact of PLX51107-mediated BET inhibition on FcγR expression and FcγR-mediated inflammation is unknown. As such, we treated healthy human monocytes with incremental concentrations of PLX51107 for 24 h and tested whether FcγR transcript expression was altered using quantitative real-time PCR (qPCR; Figure 1A–E). We used concentrations from 0 to 250 nM, as PLX51107 had been shown to downregulate expression of certain proteins at 100 nM [13]. The 24 h time period was sufficient to observe any apoptotic effects [13] and to permit differences to surface with regard to FcγR protein expression due to turnover. Longer time periods were deemed unreliable due to the known loss of viability starting at two days of culture [14,15] as well as potential changes in phenotype, such as those seen in classical monocytes after 24 h in circulation [16].

Results showed that treatment with PLX51107 significantly downregulated FcγRIIa, FcγRIIb, and FcγRIIIa mRNA expression (Figure 1A–C). Interestingly, mRNA expression of FcγRIa was upregulated with increasing concentrations of PLX51107 (Figure 1E); however, upon further investigation, it was determined that FcεR1γ transcript expression (γ-chain) was also diminished upon treatment with PLX51107 (Figure 1D).

**Figure 2:**
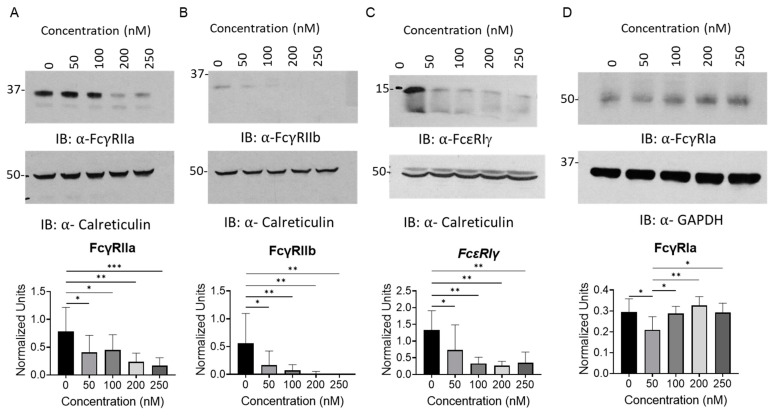
BET inhibition reduces FcγR protein expression in human monocytes. Healthy-donor CD14^+^ monocytes were cultured for 24 h with PLX51107 (50, 100, 200, 250 nM) or with vehicle control (DMSO) for 24 h. Whole cell lysates were then obtained, and protein expression was measured by immunoblotting (IB). Expression of (**A**) FcγRIIa, (**B**) FcγRIIb, (**C**) common Fc γ chain (FcεRIγ), and (**D**) FcγRIa were analyzed. Top panels show a representative western blot and densitometry quantifications are shown in the bottom panels. Calreticulin or GAPDH were used as loading controls (n = 5, n = 3 for FcγRIa). * *p* ≤ 0.05, ** *p* ≤ 0.01, *** *p* ≤ 0.001.

**Figure 3:**
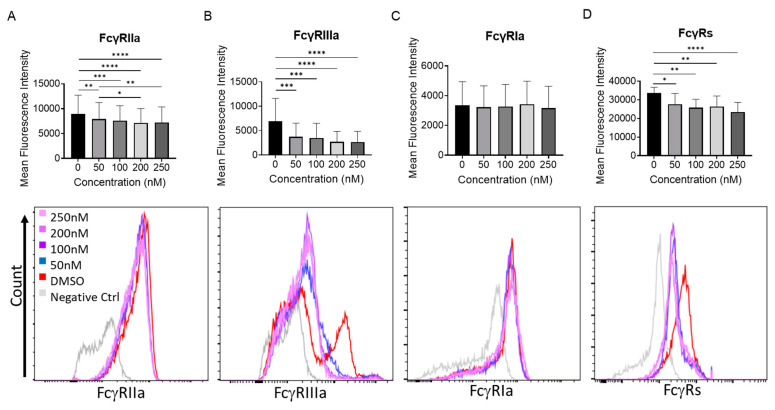
BET inhibition reduces FcγR surface expression in healthy human monocytes. Healthy-donor CD14^+^ monocytes were cultured for 24 h with PLX51107 (50, 100, 200, 250 nM) or with vehicle control (DMSO) for 24 h. Then surface expression of (**A**) FcγRIIa, (**B**) FcγRIIIa, (**C**) total FcγRs and, and (**D**) FcγRIa was quantified by flow cytometry. Top panels show mean fluorescence intensities (n = 4), while bottom panels show representative histograms. * *p* ≤ 0.5, ** *p* ≤ 0.01, *** *p* ≤ 0.001, **** *p* ≤ 0.0001 respectively.

Since FcγRIa cannot be surface expressed or signal without the γ-chain [17], it is expected that the overall FcγR response would be diminished despite the increase in the expression of FcγRIa. In order to conclusively determine whether FcγR protein and surface expression were being regulated similarly to their mRNA levels, both immunoblotting and flow cytometry analyses were conducted on healthy human monocytes with incremental dosages of PLX51107 for 24 h (Figure 2 and Figure 3). Consistent with the qPCR analyses, FcγRIIa, FcγRIIb, and γ chain protein expression was reduced, while FcγRIa protein levels did not decrease except upon 50 nM PLX51107 treatment (Figure 2D). Inhibition of BET proteins by PLX51107 also caused a modest reduction in surface FcγRIIa and FcγRIIIa, as measured by flow cytometry. Furthemore, total surface FcγR expression was decreased upon PLX51107 treatment (Figure 3D).

### 2.2. BET Inhibition Downregulates Fcγ Receptor-Mediated Signaling

**Figure 4:**
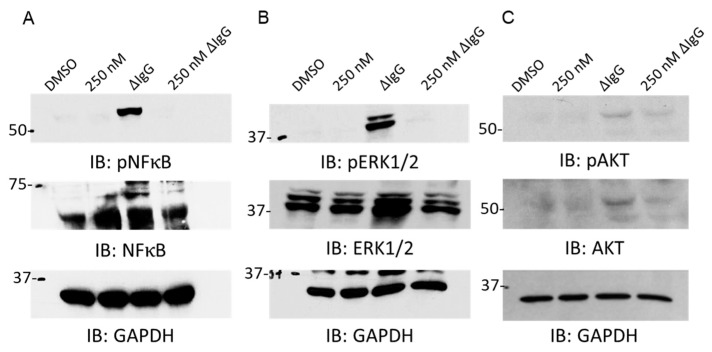
BET inhibition downregulates FcγR-mediated signaling. Healthy-donor CD14^+^ monocytes were treated with PLX51107 (250 nM) or vehicle control (DMSO) for 24 h and then subsequently activated with heat-aggregated IgG for 7 min. Whole cell lysates were obtained, and immunoblotting was done to detect activation of the (**A**) NK-κB, (**B**) MEK1/2 and (**C**) PI3K pathways with phospho-specific antibodies. GAPDH was used as loading controls. Representative western blots are shown (n = 3).

The transcription factor NF-κB plays a critical role in inducing inflammatory gene transcription, and it is potently activated in RA synovium and endothelium, particularly in CD14^+^ monocytes [18]. In order to determine if FcγR-mediated NF-κB signaling was impacted by BET inhibition, we treated healthy human monocytes with PLX51107 for 24 h, briefly stimulated the cells with ΔIgG to activate FcγRs, and isolated whole cell lysates. PLX51107 treatment was able to effectively abrogate FcγR-mediated NF-κB activation (Figure 4A). These results suggest that BET inhibition via PLX51107 treatment may be able to attenuate other FcγR-mediated signaling pathways. In order to examine if BET inhibition was able to diminish signaling pathways that are aberrantly activated in RA pathogenesis, we treated healthy human monocytes in an identical fashion as above and measured FcγR-mediated MAPK and AKT signaling [19]. Both of these signaling pathways that are characteristically found activated in RA were diminished upon PLX51107 treatment (Figure 4B,C).

### 2.3. BET Inhibition Attenuates Monocyte FcγR Function

**Figure 5:**
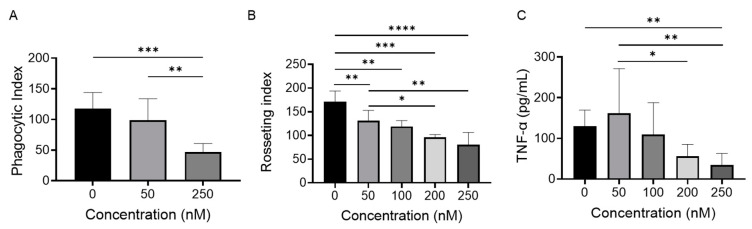
BET inhibition attenuates monocyte FcγR function. Healthy-donor PBMs were treated with PLX51107 for 24 h and then analyzed for antibody-mediated functions. (**A**) Treated CD14^+^ monocytes were incubated with IgG-opsonized sheep RBCs (SRBCs). Phagocytic index was measured using microscopy and defined as the total number of ingested RBCs per 100 CD14^+^ monocytes (n = 5). (**B**) PLX51107 treated CD14^+^ monocytes were incubated on ice with IgG opsonized SRBCs. Rosetting index was measured using microscopy and defined as total number of surface embedded RBCs per 100 CD14^+^ monocytes (n = 4). (**C**) After BET inhibition, PBMs were stimulated with heat-aggregated IgG for additional 24 h; supernatants were collected and TNFα levels were measured by ELISA (n = 5). * *p* ≤ 0.05, ** *p* ≤ 0.01, *** *p* ≤ 0.001, **** *p* ≤ 0.0001 respectively.

Having determined that BET inhibition by PLX51107 is able to regulate both FcγR mRNA, protein expression, and signaling, we next tested whether this receptor regulation had downstream functional consequences. Here, we measured three FcγR-mediated events, phagocytosis, rosetting, and TNFα production, after PLX51107 treatment in healthy human monocytes (Figure 5). CD14^+^ monocytes from healthy human donors were isolated as described above and were treated with incremental dosages of PLX51107 for 24 h, and phagocytosis was evaluated. Healthy human monocytes treated with only the vehicle were able to phagocytose SRBCs to a significantly greater extent than PLX51107 treated monocytes (Figure 5A). Rosetting was also scored using microscopy, and, similarly, PLX51107-treated monocytes exhibited significantly less rosetting compared to vehicle controls (Figure 5B). To measure FcγR-mediated production of cytokines, PLX51107-treated healthy donor monocytes were incubated for 24 h with heat-aggregated IgG (ΔIgG) in the presence of polymyxin B. Following the incubation period, supernatants were collected and TNFα levels were evaluated by ELISA (Figure 5C). There was a significant reduction in TNFα production with PLX51107 treatment.

### 2.4. BET Inhibition Regulates Fc𝛾 Receptor Expression and Function in Monocytes from Rheumatoid Arthritis Patients

**Figure 6:**
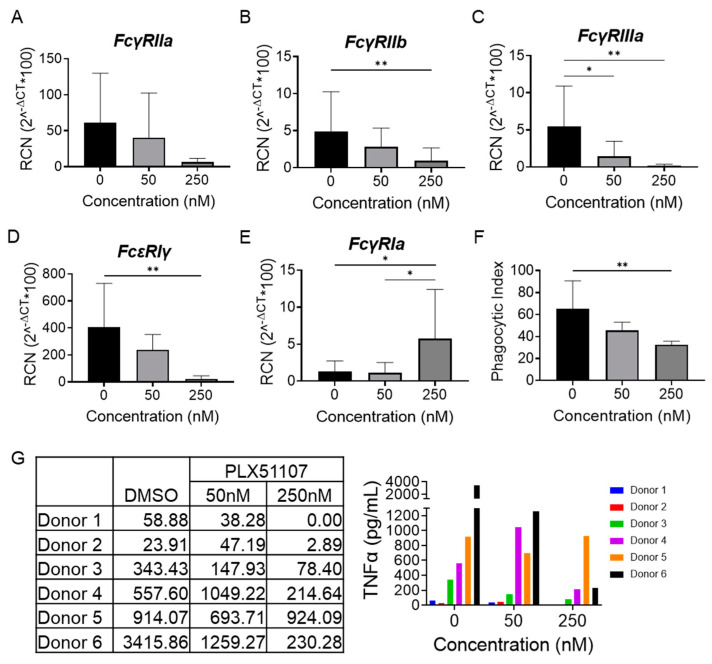
BET inhibition regulates FcγR expression and function in monocytes from rheumatoid arthritis patients. CD14^+^ monocytes from RA patients were treated with PLX51107 (50 nM, 250 nM) or vehicle control (DMSO) for 48 h and mRNA expression of (**A**) FcγRIIa, (**B**) FcγRIIb, (**C**) FcγIIIa, (**D**) FcεRIγ, and (**E**) FcγRIa, was analyzed by real-time PCR (n = 6). To analyze antibody-mediated functions, 24-h, PLX51107-treated RA patient CD14^+^ monocytes were (**F**) incubated at 37 °C with IgG opsonized sheep RBCs (SRBCs). Phagocytic index was measured using microscopy and defined as number of ingested RBCs per 100 monocytes (n = 4). (**G**) Similarly, PLX51107-treated RA patient CD14^+^ monocytes were stimulated with plate-bound IgG for another 24 h; supernatants were collected and TNFα levels were measured by ELISA; TNFα levels in control (DMSO) or PLX51107 conditions are shown for 6 different patients, represented as individual values (left) and a graph (right). * *p* ≤ 0.05, ** *p* ≤ 0.01 respectively.

To determine if BET inhibition via PLX51107 could alter FcγR transcript expression in RA patient samples, peripheral blood monocytes were isolated from RA patients and were treated with incremental dosages of PLX51107 for 24 h. Following the incubation period, qPCR was conducted to measure the expression of FcγRIa, FcγRIIa, FcγRIIb, FcγRIIIa, and the γ chain (Figure 6A–E). Similar to the results seen in healthy donor monocytes, FcγRIIa, FcγRIIb, FcγRIIIa, and γ chain expression was reduced with increasing concentrations of PLX51107, while FcγRIa expression levels were increased. To examine if BET inhibition mediated attenuation of phagocytosis and TNFα production, monocytes from RA patients were isolated, treated, and functionally assessed as described above (Figure 6F). Results showed that both phagocytosis and production of the potently upregulated inflammatory cytokine TNFα characteristic of RA were significantly diminished upon treatment with PLX51107.

### 2.5. BET Inhibition Reduces Footpad Inflammation and FcγR Expression In Vivo

**Figure 7:**
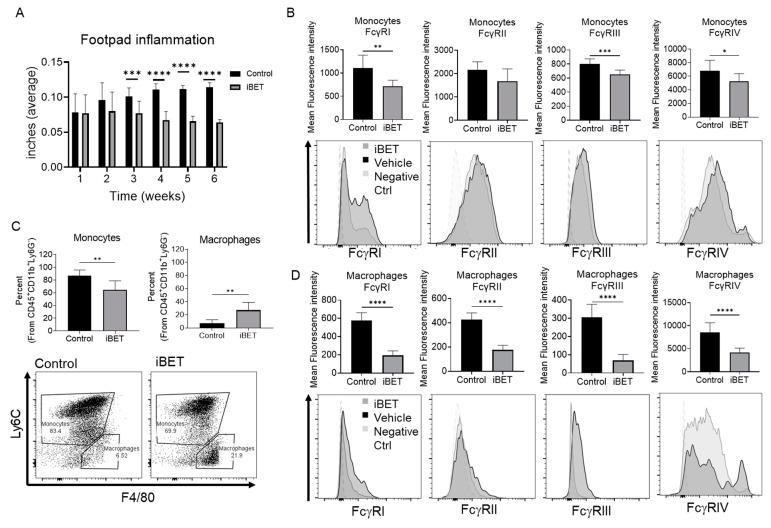
BET inhibition reduces footpad inflammation and FcγR expression in vivo. DBA mice were injected with collagen type II as indicated in the methods to induce rheumatoid arthritis. After 2 weeks from the last collagen injection, mice were treated with vehicle or PLX51107 (iBET) at 10mg/kg three times a week for 5 weeks. During the treatment, (**A**) footpad swelling was measured every week, showing the average between left and right front foot (n = 10 per group). Mice were then sacrificed 2 weeks after the last treatment, spleens obtained and used to detect expression of FcγRs in (**B**) monocytes, and (**D**) macrophages, as well as the percentage of (**C**) monocytes and macrophages. Graphs show the mean fluorescence intensities, as well as representative histograms below. * *p* ≤ 0.05, ** *p* ≤ 0.01, *** *p* ≤ 0.001, **** *p* ≤ 0.0001 respectively.

To elucidate the role of BET inhibition in FcγR expression in monocytes and macrophages in vivo, we used the CIA mouse model, similar to what has been reported in the literature [20]. After disease induction, mice were treated with vehicle or PLX51107 three times a week, for a total of 16 doses. During treatment, measurements of the left and right footpads were taken (Figure 7A). As shown, the average footpad thickness was significantly reduced three weeks after starting BET inhibitor treatment in comparison to the vehicle. This statistically significant difference was also seen at weeks 3 to 5 of the treatment, and it remained even after treatment has been stopped at week 6.

To verify the effects of BET inhibition on FcγR in monocytes and macrophages in vivo, we measured the expression of the receptors in these cells using the same mouse model. Two weeks after treatment ceased, mice were sacrificed, and the spleens were obtained. Surface expression of FcγR was measured using flow cytometry (Figure 7B,D), marking monocytes as CD45^+^CD11b^+^Ly6G^−^LyC6^+^. Results showed that FcγRI, FcγRIII and FcγRIV (mouse FcγRIV corresponds to human FcγRIIIA [21,22]) were downregulated in monocytes after BET inhibitor treatment (Figure 7B). In addition, all FcγR were significantly downregulated after treatment compared to the control in macrophages (identified as CD45^+^CD11b^+^Ly6G^−^LyC6^−^F4/80^+^; Figure 7D).

Interestingly, not only the expression of FcγR was lower in treated mice, but the percentages of monocytes and macrophages were also significantly reduced in the spleen after treatment (Figure 7C, left graph). Conversely, the proportion of Ly6C^−^F4/80^+^ macrophages was increased (Figure 7C, right graph).

## 3. Materials and Methods

### 3.1. Peripheral Blood Monocyte Isolation

Human peripheral blood monocytes were isolated from either healthy donor whole blood (Versiti Blood Service of Ohio, Columbus, Ohio, USA) or from rheumatoid arthritis patients. Participation of RA patients was in accordance with an approved institutional review board protocol at Ohio State University. Mononuclear cells were obtained by density gradient using the cell separation medium (Gibco, ThermoFisher Scientific, Waltham, MA, USA). Following mononuclear cell isolation, cells were incubated with anti-CD14-coated magnetic beads (Miltenyi Biotec, Gaithersburg, MD, USA) and CD14-positive cells were collected by positive selection. Isolated monocytes were resuspended in RPMI medium supplemented with 10% fetal bovine serum (FBS), 2 mM L-glutamine, 56 U/mL penicillin, and 56 μg/mL streptomycin.

### 3.2. Cell Culture Treatments

PLX51107 (100 μM) was purchased from Selleck Chemicals (Houston, TX, USA) and was utilized to inhibit BET proteins. The inhibitor was used in concentrations of 50 nM, 100 nM, 200 nM, and 250 nM in cell culture, and dimethyl sulfoxide (DMSO) was used as the vehicle control. Human heat-aggregated IgG (ΔIgG) was prepared as described previously [23]. Briefly, 350 μg/mL of chromopure whole human IgG (Jackson ImmunoResearch Laboratories, West Grove, PA, USA) was incubated and shaken at 63 °C in a thermomixer for 90 min to allow aggregation, and immediately placed at 4 °C until use. IgG stimulation was performed in the presence of Polymixin B at 10 µg/mL (Calbiochem, Burlington, MA, USA).

### 3.3. Immunoblotting

Immunoblotting was conducted as described previously [23,24]. Healthy peripheral blood monocytes were lysed using TN1 buffer (50 mM Tris (pH 8.0), 10 mM EDTA, 10 mM Na_4_P_2_O_7_, 10 mM NaF, 1% Triton X-100, 125 mM NaCl, 10 mM Na_3_VO_4_, and 10 μg/mL each of aprotinin and leupeptin) for 30 min. Protein concentrations were quantified using the detergent compatible protein assay kit (Bio-Rad Laboratories, Hercules, CA, USA), and lysates were subsequently boiled with 5× Laemmli sample buffer, containing 10 µL/mL of β-Mercaptoethanol. Proteins were then separated using SDS-PAGE, and transferred onto nitrocellulose membranes (Bio-Rad Laboratories) using the Trans-Blot Turbo transfer system (Bio-Rad Laboratories). Membranes were blocked using the 5% blotting grade blocker (Bio-Rad Laboratories) and subsequently probed with the appropriate primary and anti-rabbit-HRP secondary antibodies. Membranes were developed using the Pierce™ ECL Western Blotting Substrate or SuperSignal^®^ West Femto Maximum Sensitivity Substrate (ThermoFisher Scientific, Waltham, MA, USA). Protein abundance in the blots was quantified via densitometric analysis using Image J software (National Institutes of Health, Bethesda, MD, USA), and the abundance of the protein of interest was compared against that of the loading control, calreticulin, or GAPDH. The following antibodies were utilized: anti- FcγRIa, anti-FcγRIIa, anti-FcγRIIb (Abcam/Epitomics, Cambridge, UK), anti-FcεRI γ-subunit (MilliporeSigma, Burlington, MA), anti-phospho-NF-κB p65 (Ser536), anti-NF-κB, anti-phospho-p44/42 MAP Kinase (Thr202/Tyr204), anti-p44/42 MAPK, anti-phospho-Akt (Ser473), anti-AKT, anti-GAPDH (Cell Signaling Technology, Danvers, MA, USA), anti-calreticulin (Enzo Life Sciences, Farmingdale, NY, USA), and anti-rabbit IgG-HRP (Santa Cruz Biotechnology, Dallas, TX, USA).

### 3.4. Phagocytosis

Phagocytosis assays were performed as described previously [24]. Sheep red blood cells (SRBCs, Colorado Serum Company, Denver, CO, USA) were labeled with PKH26 fluorescent cell membrane dye (Sigma-Aldrich, St. Louis, MO, USA) and then subsequently opsonized with anti-SRBC antibody (Sigma-Aldrich). 1 μL of gently pelleted SRBCs were added to the respective healthy peripheral blood monocytes or rheumatoid arthritis peripheral blood monocytes, and then incubated in the dark at 37 °C for 1 h. Non-phagocytosed SRBCs were briefly lysed with water, followed by 1% paraformaldehyde fixation. The SRBCs ingested by 100 monocytes were scored in a blinded fashion using fluorescence microscope Olympus DP71 (Evident/Olympus, Tokyo, Japan), with two separate counts per experimental treatment group. Phagocytic index was measured and defined as the total number of SRBCs ingested by 100 monocytes.

### 3.5. Rosetting

SRBCs (Colorado Serum Company) were labeled with PKH26 fluorescent cell membrane dye (Sigma-Aldrich) and then subsequently opsonized with anti-SRBC antibody (Sigma-Aldrich) as described above. 1 μL of gently pelleted SRBCs were added to the respective healthy peripheral blood monocytes, and then incubated in the dark on ice for 1 h, followed by 1% paraformaldehyde fixation. The SRBCs embedded on the surface of 100 monocytes were scored in a blinded fashion using fluorescence microscope Olympus DP71, with two separate counts per experimental treatment group. The rosetting index was measured and defined as the total number of SRBCs embedded on the surface by 100 monocytes.

### 3.6. Real-Time Polymerase Chain Reaction (qPCR)

Real-time PCR was carried out as previously described [24]. RNA was isolated from healthy and rheumatoid arthritis peripheral blood mononuclear cells using the Norgen Biotek Total RNA Purification Kit (Thorold, ON, Canada) according to the manufacturer’s given instructions. RNA was reverse transcribed to cDNA using a high-capacity cDNA transcription kit (Applied Biosystems, Waltham, MA, USA) and subjected to quantitative real-time PCR using SYBR Green Master Mix (Applied Biosystems). The following human primers (Invitrogen, Waltham, MA, USA) were utilized: GAPDH (forward primer, 5′ATT CCC TGG ATT GTG AAA TAG TC-3′; reverse primer, 5′-ATT AAA GTC ACC GCC TTC TGT AG-3′), FcγRIa (forward primer, 5′-GGC AAG TGG ACA CCA CAA AGG CA-3′; reverse primer, 5′- GCT GGG GGT CGA GGT CTG AGT-3′), FcγRIIa (forward primer, 5′-TTG CTG CTG CTG GCT TCT GC-3′; reverse primer, 5′-GTA GCT GGG CTG CGT GTG GG-3′), FcγRIIb (forward primer, 5′-TGA CTG CTG TGC TCT GGG CG-3′; reverse primer, 5′-AGC CTT TGG GGG AGC AGG TGT-3′), FcγRIIIa (forward primer, 5′-CCT CTC CAC CCT CAG TGA CCC G-3′; reverse primer, 5′-TGG AGC AAC AGC CAG CCG AT-3′), and FcεRIγ (forward primer 5′-CAA GCA GCG GCC CTG GGA G-3′and reverse primer 5′-TTC CTG GTG CTC AGG CCC GT-3′). GAPDH was used for normalization of the genes of interest. Data were presented as mean relative copy number, and were relative copy number (RCN) = 2−ΔCt × 100, where ΔCt was the difference in cycle threshold (Ct) between the gene target and GAPDH.

### 3.7. Enzyme Linked Immunosorbence Assay (ELISA)

ELISAs were conducted as previously described [25]. Supernatants from healthy and rheumatoid arthritis donor cell cultures treated with PLX51107 (50 nM, 100 nM, 200 nM, 250 nM) or the vehicle, dimethyl sulfoxide (DMSO, 250 nM), were collected after 48 h using the Human TNFα DuoSET ELISA kit (R&D Systems, Minneapolis, MN, USA) utilizing manufacturer provided instructions. To measure FcγR-mediated TNFα production, both healthy- and RA-donor cell cultures were treated with 350 μg/mL heat aggregated IgG (ΔIgG) in the presence of 1 μL of polymyxin B for 24 h.

### 3.8. Fc𝛾 Receptor Flow Cytometry

To study the effects of BET inhibition on FcγR surface expression, healthy peripheral blood monocytes were collected 24 h after treatment and washed two times in PBS. Cells were then incubated with mouse F(ab’)_2_ anti-FcγRIa (clone 32.2), FcγRIIIa (clone 3g8), mouse F(ab) anti human FcγRIIa (clone IV.3), or whole human IgG for 30 min on ice. Next, cells were washed in staining buffer (0.5% BSA in PBS) and incubated with either Alexa Fluor. 647 goat anti-mouse F(ab) or Alexa Fluor. 488 goat anti-human IgG for 30 min on ice. A sample with secondary antibodies only was used as the staining control. Finally, samples were washed and analyzed using the BD Biosciences LSR Fortessa (Flow Cytometry Shared Resource, OSU) and FlowJo Software (FlowJo LLC, Ashland, OR, USA). Results are shown as mean fluorescence intensities (MFIs), calculated as the mean of the stained samples minus the secondary antibody control.

### 3.9. Collagen-Induced Arthritis Mouse Model

To elucidate the effects of BET inhibition on FcγR in vivo, we used the collagen-induced arthritis mouse model (CIA), similar to what has been described previously [20]. All animal studies were approved by the institutional animal care and use committee at Ohio State University. Nine-week-old DBA1 mice were injected intradermally at the base of the tail with 2 mg of collagen Type II (Chondrex, Inc., Woodinville, WA, USA) in combination with complete Freund’s adjuvant (Chondrex, Inc.) at 1:1 ratio. After 3 weeks, a booster with 1 mg of collagen mixed with incomplete Freund’s adjuvant (Chondrex, Inc.) at a 1:1 ratio was injected. Two weeks after the booster, treatment with vehicle (5% sodium carboxymethylcellulose in saline solution) or PLX51107 in DMSO mixed with vehicle at 10 mg/kg was given by oral gavage three times per week. Footpad inflammation was measured using a caliper every week, and treatment was given for 5 weeks, intradermally. Two weeks after the last treatment, mice were sacrificed, and spleens obtained. Spleens were minced and passed through a 100 µm mesh, centrifuged with separation media to eliminate erythrocytes, and washed. Cells were incubated with whole mouse IgG at 10 µg/mL for 15 min and then incubated with the following antibodies: Brilliant Violet αCD45 (Biolegend, San Diego, CA, USA; clone 30-F11), Brilliant Violet 510 α-CD11b (Biolegend, clone M1/70), Brilliant Violet 605 αF4/80 (Biolegend, clone BM8), PE/Cyanine7 αLy6C (Biolegend, clone HK1.4), PerCP αLy6G (Biolegend, clone 1A8), APC αCD64/FcγRI (Biolegend, clone S18017D), Alexa Fluor 700 αCD32/FcγRII (Biolegend S17012B), PE αCD16.2/FcγRIV (Biolegend, clone 9E9), and Alexa Fluor 488 αCD16/FcγRIII (Novus Biologicals, Centennial, CO, USA; clone 275003). Zombie NIR viability staining was also used (Biolegend). Cells were incubated in ice for 30 min, washed, and fixed with 2% paraformaldehyde before acquisition.

### 3.10. Statistical Analysis

For Figure 1, Figure 2, Figure 3, Figure 4, Figure 5, Figure 6 and Figure 7A, analysis was performed using the SAS software (SAS Institute, Cary, NC, USA), by Dr. Xiaokui Mo, from the Department of the School of Biomedical Sciences at Ohio State University. For Figure 1, Figure 2, Figure 3, Figure 4, Figure 5 and Figure 6, statistical analysis was conducted using the mixed effects model with a trend analysis. For Figure 7A, analysis was conducted using the mixed effects model followed by comparisons between every time point, and trend analysis was also included. For Figure 7B–D, unpaired two tail *t*-test analysis used GraphPad Prism software.

## 4. Conclusions

Epigenetic DNA modifications are of great importance to modify the expression of proteins regulating cellular processes such as inflammation. In autoimmune diseases, and especially in RA, epigenetic regulation has been linked to the pathology of the disease [26]. Thus, the use of transcription modifiers has tremendous therapeutic potential in inflammatory diseases.

Among therapeutically relevant epigenetic regulators are the BET family of proteins. The BET family includes four bromodomain containing proteins: BRD2, BRD3, BRD4, and BRDT [11]. These bromodomain containing proteins are found among a wide variety of species, and are capable of interacting with acetylated histones and thus initiate and promote active transcription [11]. BET inhibitors are currently used in preclinical and clinical trials to demonstrate their safety and efficacy against malignant diseases [27]. Of relevance, it was initially shown that BET inhibition resulted in reduced inflammatory response in bacteria-induced sepsis [12]. For these reasons, BET inhibitors are attractive therapeutic agents for RA [28].

Here, we have demonstrated the key role that the BET inhibitor, PLX51107, plays in modulating the expression of FcγR in both healthy and RA CD14^+^ monocytes. Since FcγR1a and FcγRIIIa signal through the γ-chain, their function is depleted irrespective of their presence on the cell surface. Indeed, other studies have found that the γ-chain is necessary for FcγRIIIa expression and signal transduction, and further demonstrated that mice that lack the γ-chain were protected from autoimmune arthritis and other forms of immune damage [29]. In addition, studies using serum-induced RA mouse model demonstrated that FcγRI expression does not affect disease progression [30]. On the other hand, reduction in FcγRIIa may be of extreme importance, as previous studies have found that macrophages produce TNFα when FcγRIIa engages IgG ACPA complexes [31]. Additionally, RA monocytes display more surface FcγRIIa than healthy control monocytes, further corroborating the relevance of PLX51107 reducing FcγRIIa expression in RA monocytes [32].

Intracellular inflammatory kinase signaling pathways play a key role in RA pathogenesis and progression and are further dysregulated by the presence of inflammatory cytokines, such as TNFα and IL-1β [19,33]. Indeed, JAK inhibitors, such as tofacitinib, have shown efficacy in reducing autoimmune inflammation [19]. BRD4 is able to recruit additional transcription factors to NF-κB-dependent promoter regions upon inflammation-induced acetylation of the RELA subunit of NF-κB, further upregulating NF-κB inflammatory signaling cascades [34]. In RA, p65 and p50 have been shown to be present in CD14^+^ cells in the synovium [35]. Further, NF-kB activation may be correlated with hyperplasia and pannus formation [18]. The blockade of BRD4, and, in turn, the blockade of NF-kB, may limit the inflammatory process in the synovium and reduce joint damage.

We have also shown that PLX51107 treatment is able to mitigate FcγR-mediated inflammation, as seen through the attenuation of TNFα production, phagocytosis, and FcγR-mediated signaling pathway activation. The reduction in phagocytosis and inflammatory cytokine production by healthy donor and RA monocytes mediated by PLX51107 provide a rationale as to why BET inhibition should be further studied in the context of rheumatoid arthritis. Indeed, RA synovium and serum not only have a higher concentration of TNFα but also a higher abundance of TNFα receptors, making them particularly sensitive to this potent inflammatory cytokine [36].

To elucidate the effectiveness of PLX51107 in vivo, we used the collagen-induced arthritis mice model, as it is widely used to study RA. BET inhibition not only resulted in decreased footpad inflammation but, also, in reduced expression of FcγR in monocytes and macrophages in the spleen of treated mice compared to vehicle controls. These results provide evidence that inhibition of BET can reduce the expression of FcγR not only in vitro but also in vivo. Of relevance in the CIA mouse model is the significant decrease in FcγRIV, which has been found to be increased in osteoclasts and is related to pannus formation [37]. Thus, PLX51107-mediated FcγRIV may lower the chance of pannus formation. On the other hand, it has been reported that a lack of FcγRIIb results in increased vulnerability to CIA in resistant mice [38]. However, we see a decrease in RA progression after PLX treatment, which may be attributed to the downregulation of activating FcγR. Although we saw differences with roughly 1 month of treatments (16 treatments at 3 times per week), the question remains of whether any ill effects would surface over a longer period of time. In one treatment group examined by Ozer et al. [13] [PMID: 29386193], some mice were treated for up to 150 days with no reported effects. In addition, it is unknown whether the effects on FcγR would be sustained. Future experiments will be needed in order to test this.

In addition to reduced FcγR expression in vivo, we observed a modest but significant reduction of monocyte percentages, identified as CD45^+^CD11b^+^Ly6G^−^Ly6C^+^ cells, while there was an increase in the number of macrophages, identified as CD45^+^CD11b^+^Ly6G^−^Ly6C^−^F4/80^+^ cells. In inflammatory responses, Ly6C^high^, or classical monocytes, are known to accumulate in inflammation sites such as atherosclerotic plaques [39]. Interestingly, previous work has shown that in the absence of classical monocytes, RA-related bone erosion is increased, as well as the percentage of non-classical monocytes in peripheral blood [40]. However, no studies so far have correlated these observations with other secondary inflammatory sites, such as the spleen.

The increase in F4/80^+^Ly6C^−^ macrophages in the spleen may be indicative of a less inflamed state in treated mice. Depending on the polarization state, macrophages can help resolve inflammation and promote tissue repair after injury [41]. Since the percentage of total monocytes and macrophages observed in our RA model remains stable among treatments, we hypothesize that Ly6C^+^ monocytes may differentiate into F4/80^+^ macrophages, as described previously, and may be indicative of a less inflamed state [39]. Further studies are required to test this hypothesis by elucidating the phenotype of macrophages after PLX51107 treatment.

In conclusion, our studies presented here offer a novel method to decrease inflammation in rheumatoid arthritis. While BET inhibitors have been studied in the context of reducing inflammatory cytokine production in RA [28], there have been no studies investigating their impact on FcγR-mediated response in RA. By mitigating FcγR-mediated inflammation, BET inhibitors are able to modulate a crucial component of rheumatoid arthritis. Hence, BET inhibitors may be a potential alternative to treating patients with RA that are difficult to manage with other standards of care.

## Data Availability

Full images of western blots have been uploaded to the MDPI as part of this submission.
